# Material composition of the mouthpart cuticle in a damselfly larva (Insecta: Odonata) and its biomechanical significance

**DOI:** 10.1098/rsos.172117

**Published:** 2018-06-13

**Authors:** Sebastian Büsse, Stanislav N. Gorb

**Affiliations:** Department of Functional Morphology and Biomechanics, Institute of Zoology, Christian-Albrechts-Universität zu Kiel, Am Botanischen Garten 9, 24118 Kiel, Germany

**Keywords:** Zygoptera, prehensile labial mask, feeding biomechanics, confocal laser scanning microscopy, finite-element modelling

## Abstract

Odonata larvae are key predators in their habitats. They catch prey with a unique and highly efficient apparatus, the prehensile mask. The mandibles and maxillae, however, play the lead in handling and crushing the food. The material composition of the cuticle in the biomechanical system of the larval mouthparts has not been studied so far. We used confocal laser scanning microscopy (CLSM) to detect material gradients in the cuticle by differences in autofluorescence. Our results show variations of materials in different areas of the mouthparts: (i) resilin-dominated pads within the membranous transition between the labrum and the anteclypeus, which support mobility and might provide shock absorption, an adaptation against mechanical damage; (ii) high degrees of sclerotization in the incisivi of the mandibles, where high forces occur when crushing the prey's body wall. The interaction of the cuticle geometry, the material composition and the related musculature determine the complex concerted movements of the mouthparts. The material composition influences the strength, mobility and durability of the cuticular components of the mouthparts. Applying CLSM for extracting information about material composition and material properties of arthropod cuticles will considerably help improve finite-element modelling studies.

## 1. Background

Insect cuticle is a complex composite material with a great variety of material properties. It gives the insect body its shape and supports the muscles, but its multifunctional character comprises other functions as well, for example, waterproofing, temporary food storage and serving as a barrier against parasites and pathogens. Furthermore, it possesses a range of mechanical specializations, for example, high compliance, storage of elastic energy, adhesion and wear resistance in specific body regions [1–3]. These functions are facilitated by the immense variety of materials in the insect cuticle and by their fundamentally different mechanical properties. These differences become clearer by comparing Young's modulus *E*, a measure of stiffness in solid materials: (i) resilin, *E* ∼ 1 MPa, (ii) soft cuticles, *E* = 1 kPa to 50 MPa and (iii) sclerotized cuticles, *E* = 1–20 GPa [4].

**Figure 1.**
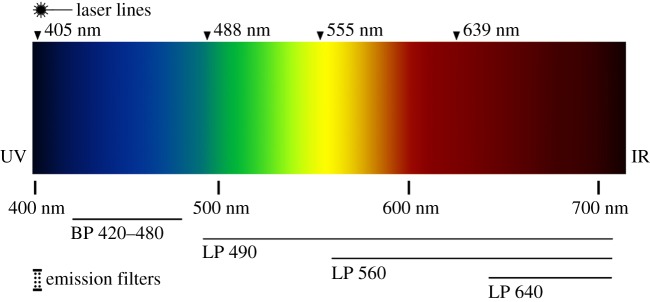
Light spectrum (visible) with wavelengths, including the laser lines and emission filters used for CLSM in this study.

Resilin is an elastic protein found in insect cuticle [5], whose amino acid composition was first analysed by Bailey & Weis-Fogh [6]. This protein has been found in the mechanical structures of many arthropods by using its blue autofluorescence when examined under ultraviolet light (405 nm, cf. figure 1) [7–13]. Resilin is found in insect cuticle wherever high resilience, low fatigue or strong damping is required. Here, the multifunctional character of resilin becomes clear: it can be stretched to up to three times its original length, and it exhibits a resilience of up to 97% and a fatigue limit of greater than 300 million cycles [5,14]. In insect wings, resilin contributes to the passive adjustment of camber and wing deformation and to the reduction of material fatigue at sites with strong or frequent deformations [7,9–11,15,16]. It is also used for storing elastic energy for subsequent high-speed movements, when muscles would not be able to move as fast as required, as, for instance, in the hind legs of cicadas [8] or locusts [12]. Burrows *et al.* [17] detected a reverse mechanism which enables the high-speed jump of froghoppers (Insecta: Cicadomorpha); the energy is stored by the chitinized cuticle, while the resilin rapidly returns the body to its original shape after a jump and allows for repeated jumping (in this case, resilin stores only 1–2% of the energy needed for the jump). Similarly, a combination of different material properties supports other biomechanical functions in arthropods, as, for example, in the mandibles of copepods. Here, strongly sclerotized and mineralized cuticle can crush diatom shells; simultaneously, the risk of mechanical damage is reduced by resilin which is integrated in the socket of each individual tooth [18].

In this study, we used confocal laser scanning microscopy (CLSM) and the autofluorescence emitted by different materials in the insect cuticle (as suggested by Michels & Gorb [13]) to study the larval mouthparts of the damselfly *Erythromma najas*. The mouthparts of immatures of dragonflies and damselflies (Odonata) are prognathous and show the typical biting–chewing style of insects. However, the larval labium is modified into a grasping device, called prehensile labial mask, which is used for capturing prey [19,20] with a special movement which is unique among insects [20–25]. The mandibles and the very flexible maxillae play the lead in sensing, handling and crushing the food [20,26]. The functional aspects of the prehensile labial mask [20–25] have previously been studied as well as the description of cuticular structures and the muscle equipment of different mouthparts [20,26,27]. However, the influence of the material composition of the cuticle on the overall biomechanical system of the mouthparts has not been studied so far.

The aim of this study was, therefore, to elucidate, for the first time, the material composition of larval damselfly mouthparts and its possible influence on their biomechanics. The following questions were asked: (i) How are different cuticle materials with varying properties distributed within the mouthparts of larval *E. najas*? (ii) How does the distribution of these different cuticles correlate with their functional significance and the underlying biomechanics in different regions of the mouthparts? (iii) How can the information obtained in this study help improve the quality of finite-element analyses?

This study will help to better understand the complex biomechanics of the feeding process in damselfly larvae and the functional importance of the different materials that compose the larval mouthparts. Furthermore, our data will help to refine finite-element analysis (or similar mechanical modelling approaches), which is becoming more and more popular in studies of insect biomechanics and evolution, and to put some of these studies in perspective [15,16,28–32] by properly identifying cuticle regions with different material properties.

## 2. Material and methods

Immature specimens of the damselfly *E. najas* (Hansemann, 1823) were collected in Kiel (Germany) in 2016. The damselflies were determined using the key of Norling & Sahlén [33] by S.B. All specimens used for CLSM were freshly frozen and stored at −70°C. The samples were washed in ethanol; dirt particles were removed by using ultrasonic cleaning (Bandeline Sonorex RK52). The samples or dissected parts were embedded in glycerine on a glass slide and covered with a coverslip prior to scanning. For visualization, a Zeiss LSM 700 (Carl Zeiss Microscopy GmbH; wavelengths 405, 488, 555 and 639 nm and emission filters BP420–480, LP490, LP560, LP640 nm) was used (figure 1). Maximum intensity projections were created using the ZEN 2008 software (www.zeiss.de/mikroskopie) and subsequently processed in Affinity Photo and Affinity Design (Serif Ltd, www.affinity.serif.com). For more information on using CLSM to determine the material properties of insect cuticle, we refer to Michels & Gorb [13].

**Figure 2.**
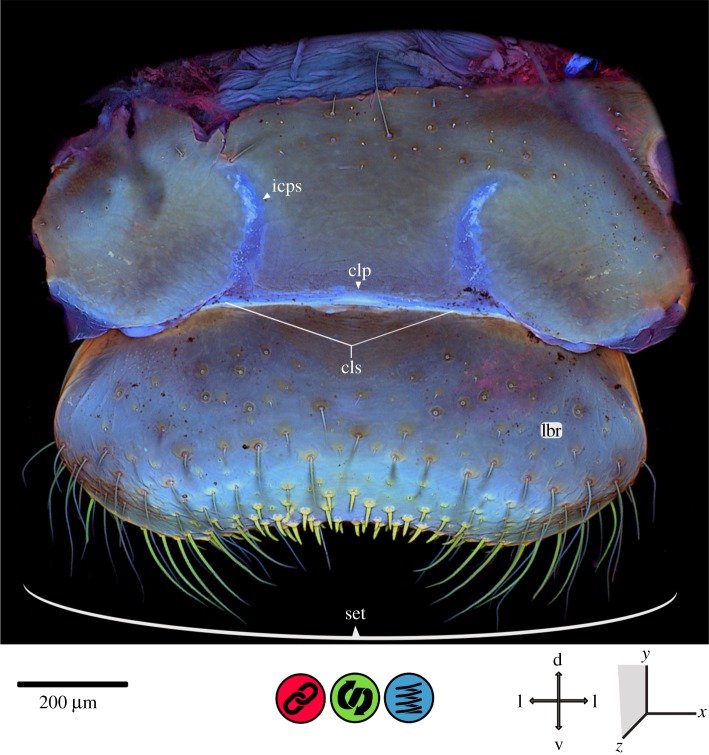
CLSM maximum intensity projection of the labrum of *Erythromma najas*, frontal view. clp, clypeolabral pad; cls, clypeolabral suture; d, dorsal; icps, intra-clypeal sutures; l, lateral; lbr, labrum; set, setae; v, ventral.

**Figure 3.**
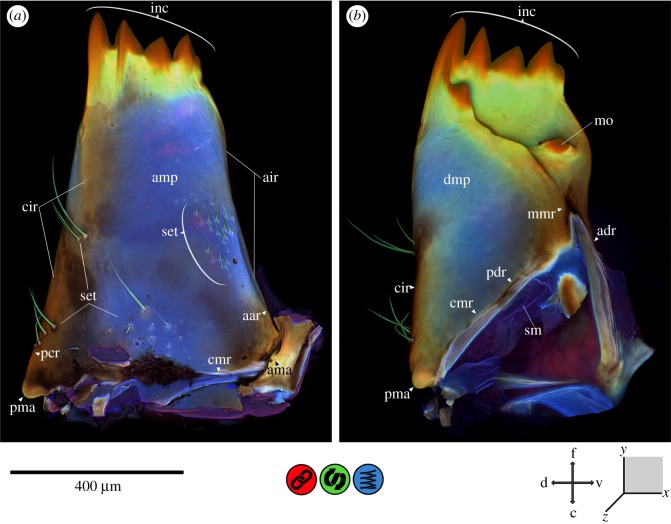
CLSM maximum intensity projection of the mandible of *Erythromma najas*. (*a*) Aboral view and (*b*) oral view. aar, anterior acetabular ridge; adr, anterior dorsal ridge; air, acetabular–incisivi ridge; ama, anterior mandibular articulation; amp, aboral mandibular pad; c, caudal; cir, condylar–incisivi ridge; cmr, circum-mandibular ridge; d, dorsal; dmp, dorsal mandibular pad; f, frontal; inc, incisivi; mmr, median molar ridge; mo, mola; pcr, posterior condylar ridge; pdr, posterior dorsal ridge; pma, posterior mandibular articulation; set, setae; sm, suspensional membrane; v, ventral.

**Figure 4.**
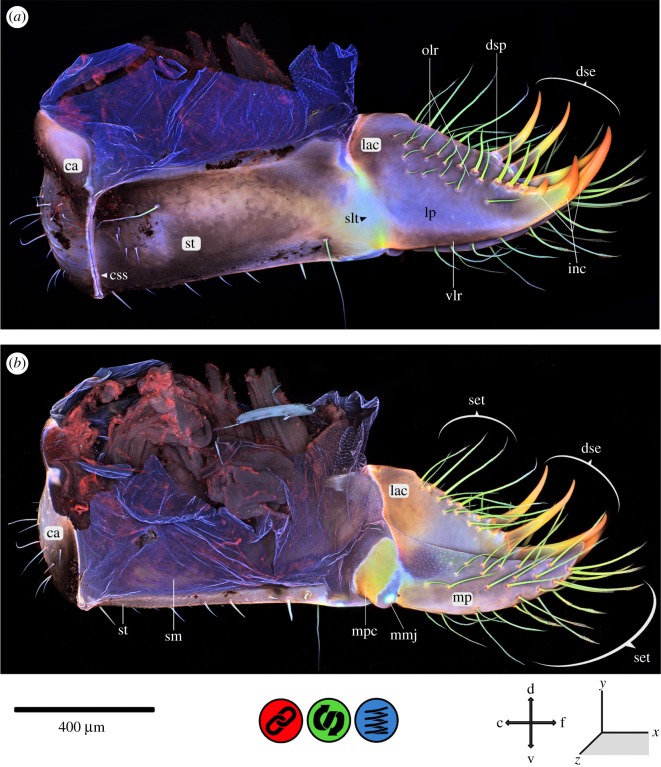
CLSM maximum intensity projection of the maxilla of *Erythromma najas*. (*a*) Lateral view and (*b*) median view. c, caudal; ca, cardo; css, cardo-stipedial suture; d, dorsal; dse, dentisetae; dsp, dentisetal pads; f, frontal; inc, incisivus; lac, lacinia; lp, lacinial pad; mmj, maxillary palpus, maxillae joint; mp, maxillary palpus; mpc, maxillary palpus clasp; olr, oral lacinia ridge; set, setae; slt, stipes–lacinia transition; sm, suspensional membrane; st, stipes; v, ventral; vlr, ventral lacinia ridge.

**Figure 5.**
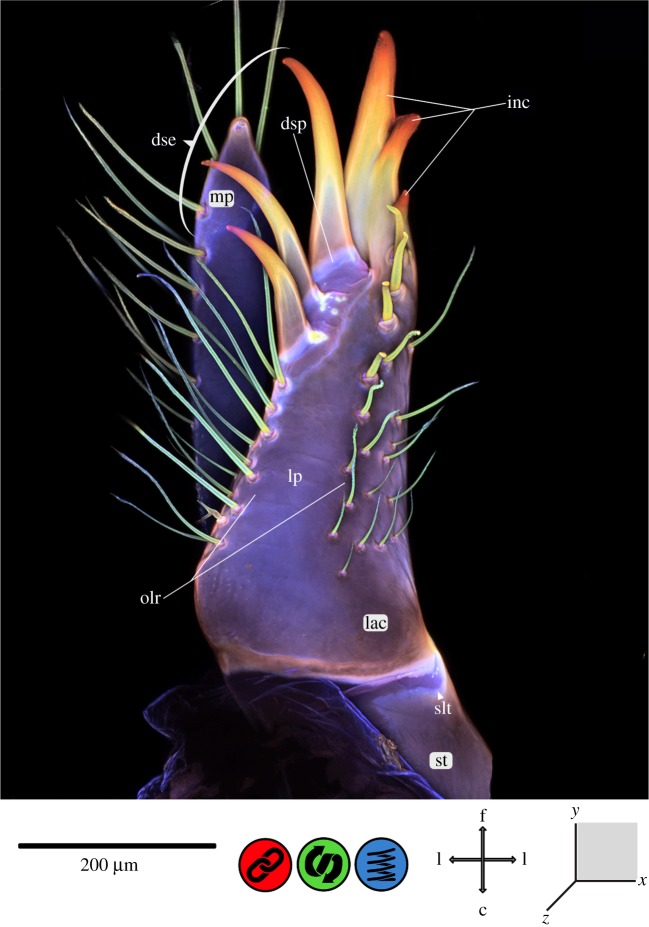
CLSM maximum intensity projection of the lacinia (part of the maxilla) of *Erythromma najas*, oral view. c, caudal; d, dorsal; dse, dentisetae; dsp, dentisetal pads; f, frontal; inc, incisivus; l, lateral; lac, lacinia; lp, lacinial pad; olr, oral lacinia ridge; slt, stipes–lacinia transition; st, stipes; v, ventral.

**Figure 6.**
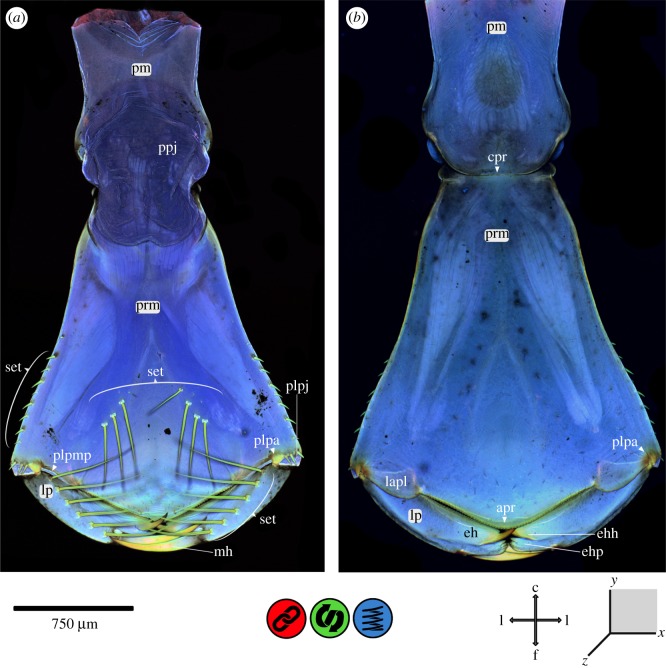
CLSM maximum intensity projection of the labium of *Erythromma najas*. (*a*) Dorsal view and (b) ventral view. apr, apico-premental ridge; c, caudal; cpr, circum-postmental ridge; eh, end hook; ehh, end hook–hook; ehp, end hook–palm; f, frontal; l, lateral; lp, labial palpus; lapl, latero-apical premental lobe; mh, movable hook; plpa, prementum–labial palpus articulation; plpj, prementum–labial palpus joint; plpmp, prementum–labial palpus membrane pad; pm, postmentum; prm, prementum; ppj, prementum–postmentum joint (p–p joint); set, setae.

**Figure 7.**
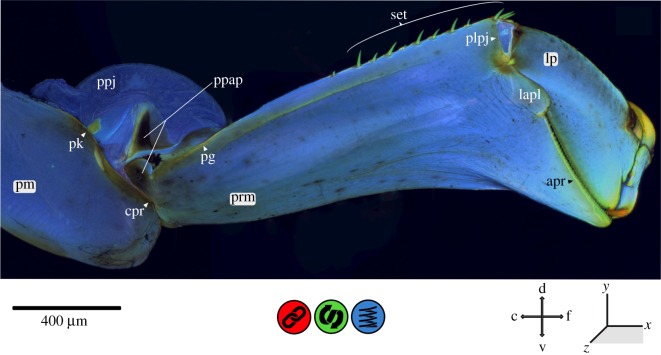
CLSM maximum intensity projection of the labium of *Erythromma najas*, lateral view. apr, apico-premental ridge; c, caudal; cpr, circum-postmental ridge; d, dorsal; f, frontal; l, lateral; lp, labial palpus; lapl, latero-apical premental lobe; pg, premental groove; pk, postmental knob; plpj, prementum–labial palpus joint; pm, postmentum; prm, prementum; ppap, prementum–postmentum articulatory plate; ppj, prementum–postmentum joint (p–p joint); set, setae; v, ventral.

**Figure 8.**
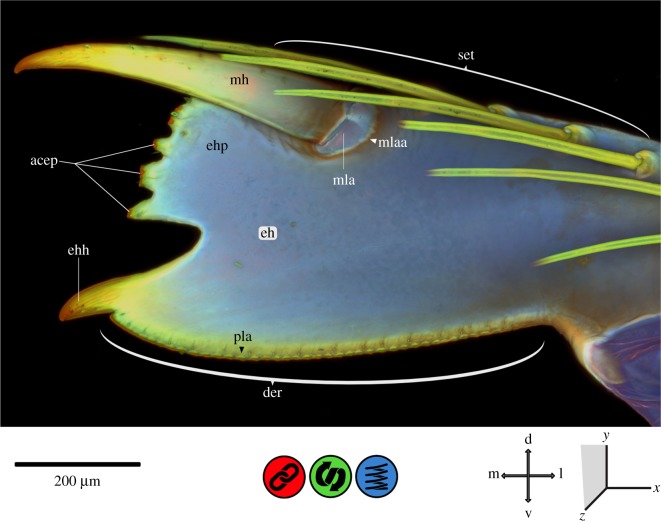
CLSM maximum intensity projection of the labial palpus (part of the labium) of *Erythromma najas*, oral view. acep, apical cap eh-paw; d, dorsal; der, dorsal eh-ridge; eh, end hook; ehh, eh-hook; ehp, eh-paw; l, lateral; m, median; mh, movable hook; mla, mh–lp articulation; mlaa, mh–lp articulation area; pla, pad-like areas; set, setae; v, ventral.

In general, insect cuticle is a composite of various components with different material properties [1,34]. The maximum intensity projections, shown in figures 2–8, illustrate the mixed autofluorescence signals coming from different materials in every single pixel [13]; as a result, it is possible to identify regions where a certain material composition dominates. According to Michels *et al.* [18], the laser excitation allows for a detailed analysis of differences in the material composition of insect cuticle. Using the material composition of the locust wing hinge (as described in [5]), the following colours can be assigned to the different autofluorescences detected (according to Michels *et al.* [18]): (i) sclerotized cuticle is characterized by red autofluorescence; (ii) relatively tough but flexible parts, consisting of chitin and resilin, show autofluorescence in blue, green and red, resulting in pink, brownish, yellow and green colours within the overlay; and (iii) very flexible, rubber-like parts with a high proportion of resilin show a characteristic light blue autofluorescence. In general, CLSM results indicate that the autofluorescence of strongly sclerotized exoskeletal parts is mainly stimulated by green to red laser light, with an emission mainly in the red part of the light spectrum, whereas less sclerotized structures show a green autofluorescence which is mainly excited by blue to green laser light. When exposed to UV or violet laser light in combination with blue laser light, the resilin-dominated structures show autofluorescence in a characteristic light blue [18, p.7].

The results of the CLSM analyses described in the following section allow for a qualitative description only and do not represent a quantitative measurement.

The aquatic immature stages of Odonata, Ephemeroptera and Plecoptera are called *naiad* following the terminological suggestions by Bybee *et al*. [35]. However, since, in Odonata, the more commonly used term is *larva*, we decided to use this more general term [36,37]. The nomenclature for morphological structures follows Beutel *et al*. [38]. The mandibular ridges or ‘pseudo’-ridges were described by Blanke *et al*. [31]. Newly introduced terms not found in the literature are defined at their first appearance in the text and put in quotation marks throughout the entire paper.

## 3. Results

The investigated mouthparts of *E. najas*—i.e. labrum (figure 2), mandibles (figure 3), maxillae (figures 4 and 5) and labium (figures 6–8)—show significant differences of their material composition as described in detail in the following and summarized in table 1.

**Table 1.** Summary of the most important mouthpart structures with information on their material composition and properties.

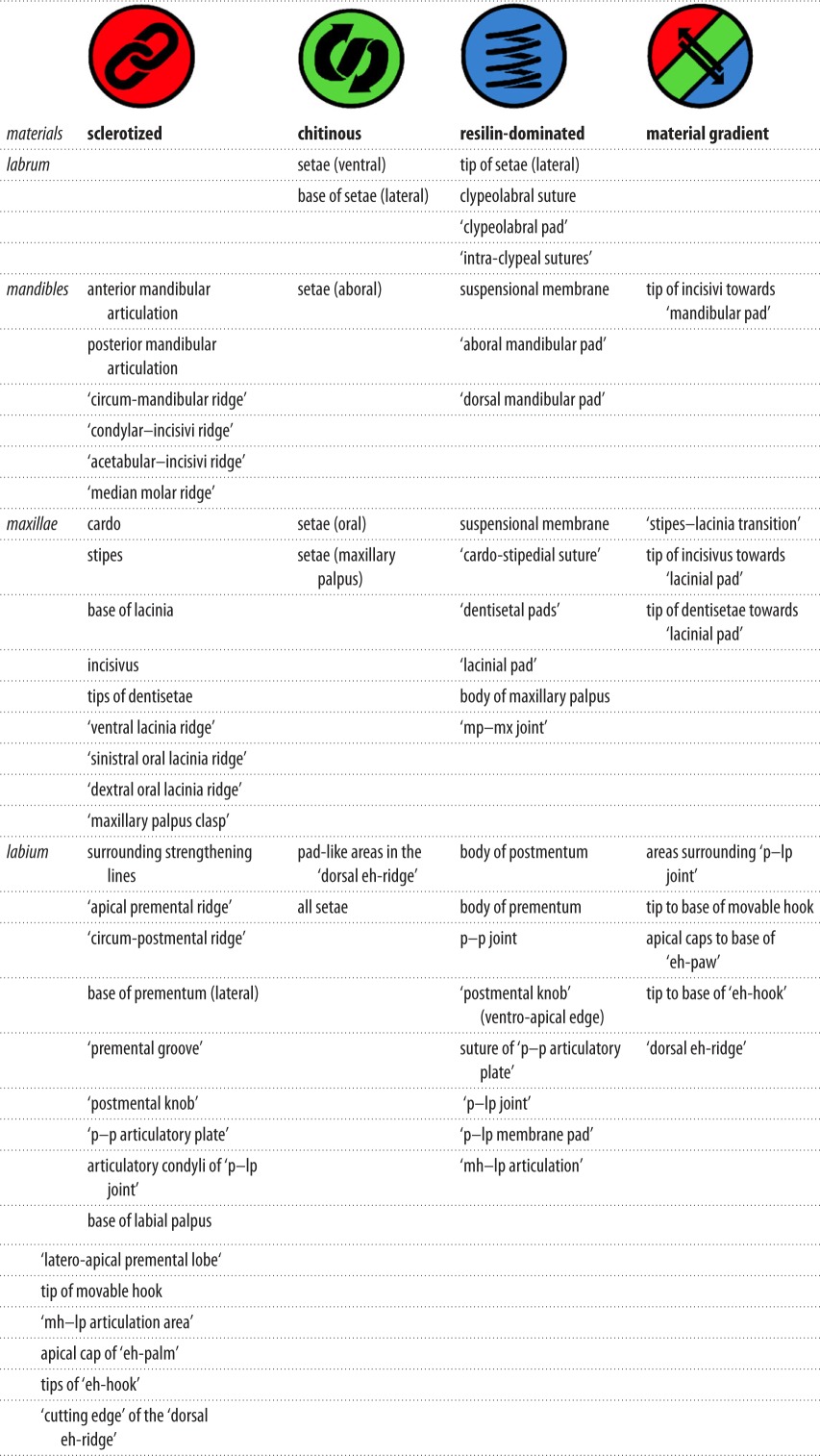

### 3.1. Labrum

The labrum is slightly kidney-shaped, and its antero-ventral part is flat and laterally extended (figure 2). It is covered with four different kinds of setae (set): (i) strong, short, chitin-dominated setae, median at the ventral surface; (ii) long, chitin-dominated setae, lateral of the median setal area; (iii) setae on the lateral surface of the labrum with chitinous cuticle at the base only and blue autofluorescence (indicating the presence of resilin) along the remaining two-thirds of their length; and (iv) short and very small, resilin-dominated setae on the frontal surface of the labrum. The ventro-median area of the labrum shows a higher amount of the viscoelastic protein resilin compared with other regions of the labrum. The clypeolabral suture (cls) is membranous. In the middle of the clypeolabral suture, a pad (‘clypeolabral pad’, clp) with considerable proportions of resilin is present. Also, resilin-dominated areas can be detected in the dorsally running sutures (‘intra-clypeal sutures’, icps) originating from the clypeolabral suture. The dorso-lateral and lateral edges of the labrum show areas with stronger sclerotization compared to the rest of the labrum.

### 3.2. Mandibles

The mandibles are composed of various materials with very different properties (figure 3). The incisivi (inc) and the tips of the molae (mo) are strongly sclerotized. There is a material gradient from the sclerotized tips with their red autofluorescence to yellow and green autofluorescence of the chitinous cuticle encircling the incisivi and molar tips. On the dorsal and aboral sides of the mandible, this material gradient continues towards more flexible, resilin-dominated areas (see below). Cranially, a strengthening line (‘circum-mandibular ridge’, cmr) encircles the internal mandibular opening. This reinforcement also comprises both the anterior (adr) and posterior dorsal ridge (pdr). At the anterior mandibular articulation (ama), the posterior condylar ridge (pcr) starts running orally; here, a strongly sclerotized strengthening line (‘condylar–incisivi ridge’, cir) follows the posterior condylar ridge and travels beyond it towards the first two—most prominent—incisivi. The same kind of strengthening line (‘acetabular–incisivi ridge’, air)—less pronounced—starts on the posterior mandibular articulation (pma) and follows the anterior acetabular ridge (aar) towards the apical incisivus (inc). Midway between the anterior and posterior mandibular articulations, on the oral surface of the mandible, a sclerotized Y-shaped strengthening line (‘median molar ridge’, mmr) is running from the ‘cmr’ towards the tip of the mandible and branches, at the height of the mola, towards the first and the last inc, respectively. Between the ‘mmr’ and the ‘acetabular–incisivi ridge’ on the one side and the ‘condylar–incisivi ridge’ on the other side, large resilin-dominated areas (‘dorsal and aboral mandibular pad’, dmp and amp) can be found. On the aboral surface of the mandible, four areas of setae (different sizes and numbers) can be identified; they are composed of chitin-dominated cuticle. Beneath the strongly sclerotized posterior and anterior mandibular articulations, the mandible is suspended by membranous areas which surround the mandibular opening.

### 3.3. Maxillae

The maxillary cuticle exhibits a mixture of very different material properties (figure 4 and 5). The cardo (ca) and the stipes (st) are sclerotized and surrounded by large membranous areas. The suture between the ca and st (‘ca-stipedial suture’, css) on the medio-lateral surface of the maxilla is composed of resilin-dominated cuticle. The suture-like transition from the st to the lacinia (‘st–lacinia transition’, slt) shows chitin-dominated areas (orally and aborally) and a resilin-dominated area (in the centre). The galea is missing; the lacinia (lac) is sclerotized at the base, with the sclerotization only interrupted by the ‘slt’ on the lateral surface of the maxilla. The inc is tripartite, with three strongly sclerotized tips, and shows a material gradient from red to yellow and greenish autofluorescence. The same red autofluorescence (characteristic of sclerotized cuticle) and the same material gradient can also be found on the dentisetae (dse) on the oral surface of the lac. The bases of the dse are encircled by strongly resilin-dominated pads (dentisetal pads). Three sclerotized strengthening lines are discernible on the maxillae: one at the back of the inc on the ventral surface of the lac (‘ventral lacinia ridge’, vlr) and two on the oral surface of the lac, forming an inverted ‘V’ with the inc at the centre (‘dextral‘ and ‘sinistral oral lacinia ridge’, olr). Between these ridges, areas with higher flexibility (‘lacinial pad’, lp) are present, indicated by bluish autofluorescence. On the oral surface of the lac, setae (composed of chitin) are present on the described strengthening lines. The maxillary palpus (mp) is also covered with setae, minute ones at the base of the palpus (dorsal surface of the maxilla) and long, prominent ones on the rest of the palpus; they all show the same chitin-dominant material composition as those on the lac. The mp is mainly composed of sclerotized cuticle. Large membranous areas are present at the connection of the mp with the maxilla itself (maxillary palpus–maxilla joint). A strongly sclerotized clasp (‘maxillary palpus clasp’, mpc) runs from dorsal to ventral (figure 4*b*). Between the ‘mpc’ and the palpus itself, resilin-dominated areas can be identified.

### 3.4. Labium

The labium (consisting of post- and prementum) is mostly composed of relatively soft and flexible cuticle (figures 6–8). The dorsal areas of the prementum–postmentum joint (p–p joint) largely consist of membranous tissues. Laterally, sclerotized ridges (strengthening lines) surround the entire pre- and postmentum: (i) a median ridge (‘apico-premental ridge’, apr) at the apical end of the prementum (between the labial palps) and (ii) a ridge running from both lateral sides dorsally to the base of the postmentum (‘circum-postmental ridge’, cpr), surrounding the p–p joint dorso-laterally. A higher degree of sclerotization is also present in lateral areas at the base of the prementum. On the ventro-lateral side of the base of the prementum, a strongly sclerotized groove is present (‘premental groove’, pg). On the corresponding area of the postmentum, a strongly sclerotized knob (‘postmental knob’, pk) is visible. Within the p–p joint articulation, a strongly sclerotized two-parted plate (‘prementum–postmentum articulatory plate’, ppap) is discernible and distinctly separated into an apical and a basal part by a resilin-dominated suture.

At the apico-lateral edge of the prementum, the labial palpus joint (p–lp joint) and the adjoining areas are strongly sclerotized in comparison to the main body of the labium. This sclerotization continues medially on the edge of a lobe (‘latero-apical premental lobe‘, lapl), which protrudes on the dorsal side of the prementum (ligula) between the ‘prementum–labial palpus joint’ (plpj) and the ‘apr’. The ‘plpj’ itself is dicondylic, consisting of a ventral and a dorsal articulatory condylus on the prementum and a counterpart on the base of the labial palpus (lp). The articulatory condyli are strongly sclerotized, with the adjoining areas showing an even stronger sclerotization—indicated by the autofluorescence that blends in a semicircular shape from a yellowish brown to green and blue. Between these condyli are large membranous areas, stretching from the apico-lateral edge of the prementum almost to the ‘apr’ (figure 6*b*). The contact areas of the ‘plpj’ membrane and the base of the lp show considerable proportions of resilin (prementum–lp membrane pad).

The lp is sclerotized at the base and at the articulatory condylus mentioned above. However, the entire labium (post- and prementum) is largely composed of softer cuticle likely supplemented with resilin. The movable hook (mh) is very prominent and strongly developed, with a material gradient that starts with red autofluorescence in the sclerotized tip, and continues with yellow and green in the middle to bluish autofluorescence at the base. At the very base, a membranous area (mh–lp articulation area) is visible, which is additionally supplemented with resilin. The areas of the mh and the lp directly adjoining and encircling the ‘mh–labial palpus articulation area’ are more strongly sclerotized than the rest of the palpus. The area of the end hook (eh) is bilobed and divided into a paw-like structure (‘eh-paw’, ehp) with 7 (5) apical blunt teeth (of which the 2./3. and 5./6. are merged) and a prominent hook (‘eh-hook’, ehh). The teeth on the ‘eh-paw’ also show a material gradient from the red autofluorescence of their sclerotized apical caps to yellow and green before merging in the blue autofluorescence of the labial palpus (figure 8). The ‘ehh’ shows the same material gradient from the strongly sclerotized tip to the resilin-dominated labial palpus. From the ‘ehh’, a dorsal saw-like ridge (‘dorsal eh-ridge’, der) runs distally towards the base of the labial palpus. The edge of this ridge is sclerotized, and pad-like areas of softer cuticle accompany the saw-like protrusions in a broken line.

The setae on the dorsal base of the prementum (including the very short ones on the apical area and on the ‘apical premental ridge’), on the dorso-lateral sides of the prementum, apically on the ‘plpj’ and on the dorsal surface of each palpus are composed of chitin-dominated cuticle.

## 4. Discussion

### 4.1. Role of the material composition in the biomechanics of the mouthparts

The *labrum* (figure 2) limits the oral cavity anteriorly; its ventral side is in direct contact with any prey consumed by the larva [20]. The relatively flexible areas of the clypeolabral suture and the epipharyngeal wall allow for the dorsal and dorso-median movements described by Büsse *et al*. [20]. Resilin-dominated areas within the clypeolabral suture considerably increase the flexibility and thus the tearing strength of the membrane [39]. The resilin-dominated areas in the ‘clypeolabral pad’ and the ‘intra-clypeal sutures’ likely provide shock absorption against mechanical impacts occurring during the feeding process. This kind of shock absorption can help achieve a uniform stress distribution which may significantly reduce the risk of structural failure [9,10,15,18,39–41]. Regions with high resilin proportions, such as those present in the labrum, are considered to improve fatigue resistance [9,10,16,18,39,40]. All this comes down to resilin's damping behaviour, to which we refer several times in the paper. Several experimental approaches have shown the considerable influence of resilin on the damping behaviour of different cuticular structures in insects [15,16,40,42]. The underpinning effect, however, is still elusive, possibly the high amount of water in resilin [5] in combination with its elastic properties allows for its damping behaviour [7,11,16,42].

The *mandibles* (figure 3) are used to crush the prey's outer body wall; they are sturdy and produce high biting forces [19,30]. The mobility of the mandibles is restricted to one axis by the mandibular articulations [20,29,31]. The material composition found in this area, i.e. the relatively strong sclerotization of the mandibular articulations and the ‘cmr’ on the one hand, and the membranous areas that surround the mandibular opening on the other hand, likely supports the mandibles' main movements of abduction and adduction along the virtual axis of rotation determined by the posterior and anterior mandibular articulation [20]. While the strongly sclerotized incisivi (as well as the mola) with their cutting edges are used for crushing the food, the other sclerotized components of the mandible, i.e. the ‘condylar–incisivi ridge’, the ‘acetabular–incisivi ridge’, the ‘mmr’ and the ‘cmr’, are responsible for wear resistance [9,10,16,18,39,40] and avoidance of structural failures [9,10,15,18,39–41] by building some kind of scaffolding and thus providing reinforcement. In between this mandibular scaffolding, large areas of resilin-dominated cuticle form the ‘mandibular pads’. These areas, in contrast, might allow for the necessary flexibility and the ability to store the stress produced by the biting forces, thus preventing structural failure [9,10,15,18,39–41]. A similar system has, for example, been described for dragonfly wings [40]. Here, the cross veins, which resemble the described mandibular scaffolding, are able to reduce the local stress by transmitting it to adjacent resilin-rich areas [40,41]. The study on copepod mandibles by Michels *et al.* [18] provides another example of the combination of different material properties in a complex biomechanical network. The copepod's strongly sclerotized and mineralized cuticles are able to crush diatom shells, while the risk of mechanical damage is reduced by flexible resilin-dominated areas integrated in the socket of each individual tooth.

The *maxillae* (figures 4 and 5) are very mobile, due to the membranous suspending areas surrounding the maxillae and the muscle arrangement [20]. The cardo and the stipes form the main body; the ‘css’ with its resilin-dominated cuticle may serve as an expansion joint to avoid damage to the maxillae.

The ‘slt’, which is composed of chitin- and resilin-dominated areas, likely allows for the abduction of the lac by the muscle 0mx6; this ability was questioned by Büsse *et al*. [20], because no true suture or joint is discernible. Instead, the flexible composite of chitin- and resilin-dominated areas seems to facilitate this abduction. Also, this transition most likely provides the necessary flexibility for coping with impact forces from the prey and thus presumably helps avoid structural failure [9,10,15,18,39–41] and lessens fatigue [9,10,16,18,39,40]. The dse are strongly sclerotized for gripping the prey and piercing its outer body wall. The prey of Odonata larvae considerably varies in size and hardness, from *Daphnia* to tadpoles (or even small fishes) [19], depending on the instar and the species. The ‘dentisetal pads’ at the base of the dse resemble bumper-like joints, likely serving to increase flexibility and thus avoid material failure [9,10,15,18,39–41]. A similar mechanism has been found, for example, in shark skin scales, where the denticles were torn out during friction tests under dry conditions, while they remained attached in wet (and therefore flexible) conditions [39]. Another more theoretical model for explaining the biomechanical function of the described material composition is the principle of mushroom-shaped adhesive microstructures (i) with joint-like narrowing and (ii) without joint-like narrowing [43].

The maxillary palpus is covered with setae which are presumably mechano- and/or chemoreceptors involved in sensing the food [44]; therefore, the palpus has a comparably softer body and is connected with the main body of the maxilla via a joint to allow for flexible movement and shock absorption.

The *labium* (figures 6–8) of damselfly larvae is transformed into a prehensile mask that allows for high-speed movement when extending the mouth towards the prey. The impact forces when hitting the prey can be considerable, because the prey often has a comparable size/weight as the predator itself (see above) [19] and may be armed with hard body walls (e.g. strongly sclerotized cuticle). The labium is composed of softer cuticle, which possibly dampens the occurring forces and reduces the impact energy transferred to the head capsule and the rest of the body [7,11,16,42].

The dorsal membranous area of the prementum-postmentum joint (p-p joint) is voluminous, with large folds on its dorsal side; if hydraulic pressure is involved in the protraction of the labial mask as currently suggested [20–25], this area may serve as a ‘pressure compensation container’ that prevents structural failure due to excessive pressure. However, the involvement of a hydraulic driving force has already been put into question by Tanaka & Hisada [24] who described structures of a click mechanism in the p–p joint, as also proposed in other, more recent publications [20,27]. In this study, we found a premental groove (‘pg’), a postmental knob (‘pk’) and a ‘prementum–postmentum articulatory plate’, which could all be involved in this click mechanism [24]. The ‘pg’ located on the ventro-lateral side at the base of the prementum and the ‘pk’ on the corresponding area of the postmentum, two corresponding and strongly sclerotized structures, are probably [24] the main components of the proposed click mechanism. The ‘prementum–postmentum articulatory plate’ and the resilin-dominated suture—both not mentioned by Tanaka & Hisada [24]—are also likely involved in this mechanism. The operation of the described click mechanism [45] is still unclear, and Tanaka & Hisada [24] did not provide any functional explanation; thus, a reconsideration of the biomechanical process of prey capturing in Odonata larvae is required.

The apical area of the prementum is in direct contact with the prey during the capturing process. The involved structures, the ‘apical premental ridge’ as well as the labial palpus with the movable hook (mh) and end hook (eh), are adapted to grasping and holding the prey, but they also need mechanisms to cope with strong impacts and high shearing forces. Beneath the ventral and dorsal articulatory condylus of the ‘plpj’, which directs the movement of the labial palpus (comparable to the dicondylic mandible in insects) and avoids shearing, the latero-apical premental lobe (‘lapl’) may help avoid a downward movement of the labial palpus and thus prevent any mechanical damage to the prementum-labial palpus joint (‘plpj’). Furthermore, the ‘prementum–labial palpus membrane pad’ that covers the base of the labial palpus and the contact area apico-laterally of the prementum might serve as a shock absorber against mechanical impact and damage due to the dampening properties of the viscoelastic resilin; in this case, the presence of resilin leads to a uniform stress distribution which can significantly reduce the risk of structural failure [9,10,15,18,39–41].

The labial palpus is moved by an adductor and an abductor muscle [20] and is able to grasp the prey directly after the extension of the prehensile mask [19]. The mh often pierces the prey and is therefore exposed to high forces during the prey capturing process. The ‘mh–labial palpus articulation area’ might be a flexible and shock-absorbing articulation, able to reduce the risk of mechanical damage or structural failure [9,10,15,18,39–41], as explained for the dentiseatae (dse) on the maxillae. The bilobed eh with the ‘hook’ and ‘paw’ structures is in direct contact with the prey. These structures show a strong apical sclerotization that might help grasp or even pierce the prey. Furthermore, the ‘apical premental ridge’ and the ‘dorsal eh-ridge’ of the labial palpus resemble a pair of scissors. The strongly sclerotized edges of these two structures can clamp the prey between the labial palpus and the prementum. Interestingly, all areas that are likely involved in contact with the prey show the same kind of strong sclerotization, such as the mandibular incisivi, maxillary dse and the structures described for the labial palpus. Furthermore, all these strongly sclerotized areas seem to be associated with resilin-dominated areas (e.g. ‘mandibular pad’ and ‘dentisetal pads’) to cope with the forces involved.

### 4.2. Implications for finite-element modelling

As mentioned in the background section, finite-element analysis (FEA), musculoskeletal modelling and other related approaches have become popular in morphological and experimental entomological contexts [15,16,28–32,40,46]. FEA is primarily used by engineers and architects to optimize the design of a model under certain boundary conditions [47,48]. Using FEA for biological structures, however, leads to other challenges [47]. The situation is reversed because the known model—the biological structure—has been optimized over millions of years of evolutionary adaptation (for Insecta, cf. [49]) and is beyond dispute. To test biomechanical or evolutionary scenarios, one needs to know the exact geometry of the components and their material properties. Within Insecta, these material properties differ immensely when compared, for example, with the endoskeleton of vertebrates [4,50].

The difference between soft cuticle (mean *E* approx. 25 MPa) and sclerotized cuticle (mean *E* approx. 10 GPa) amounts to a factor of 10^4^. Thus, by using a mean value for a structure, without information on the actual material properties (which is often a standard procedure in finite-element modelling (FEM) studies on insects), a tremendous error can be incurred.

Using CLSM for assessing the material composition, as in this study, allows for the detection of areas with different material properties and for a detailed understanding of the material distribution [3,11,18]; this knowledge can be transferred to computer models used in FEM [16,51]. Although CLSM does not allow for a precise measurement of the properties of the identified materials, the information on the material distribution can be used as a template for targeted nanoindentation or atomic force microscopy (AFM) [3,46], which in turn can be employed to refine finite-element or other models.

Nanoindentation techniques allow for the defined measurement of material properties as demonstrated by Blanke *et al*. [31] for a finite-element model of dragonfly mandibles. However, these authors found no significant differences in the measured material properties of the mandibles [31]. This cannot be supported by our findings in Zygoptera larvae. Using the measurements of Peisker *et al*. [3] on the setae of ladybird beetles and transferring them to our damselfly larvae would result in *E* = 10–20 MPa at the ‘dorsal and aboral mandibular pad’ and 6–8 GPa at the tip of the incisivi. It is difficult to determine why Blanke *et al*. [31] could not detect the material differences within the mandible found in this study, because the standard rewetting protocol [52] was used and the methodology is generally sound. However, this discrepancy illustrates the main deficiency of using nanoindentation on its own: without using CLSM as a tool for visualizing the material composition, nanoindentation alone resembles ‘blind’ testing. Thus, nanoindentation (or AFM) in combination with CLSM seems the most promising approach for three-dimensional samples [3].

Whether or not the simplification usually employed in finite-element models generally influences the obtained results is not yet known and needs to be studied in more detail. Using either CLSM or nanoindentation in combination with FEM would definitely increase the accuracy. However, combining the two techniques would allow for an unprecedented accuracy in the finite-element mapping of material properties and should become the method of choice.

In addition, sample treatment greatly affects the material properties of insect cuticle. By storage in ethanol, for instance, the Young's modulus of sclerotized cuticle is decreased by over 50% even after only 48 h [53]. The effect on soft cuticle and resilin is unknown (but the decrease is presumably even stronger). If regions with different degrees of sclerotization are affected differently by storage, the stored specimens cannot be used for elucidating the material composition with any degree of accuracy. Fresh samples are therefore the first choice for testing both material properties and material composition. If necessary, samples should be stored at −20°C or below; storage in ethanol should be avoided [47].

## 5. Conclusion

Material composition has a strong effect on the mechanics of a biological structure, as demonstrated here for damselfly mouthparts. We could show the possible structural basis for increased strength, wear resistance and shock absorption, as well as adaptations against mechanical damage and structural failure. This information is important in a biomechanical context for explaining and understanding the complexity of biological structures and the underlying functional adaptations. Thus, information on the material composition of the insect cuticle represents one of the cornerstones in basic research, which is the foundation for every biomimetic and applied approach.

Additionally, we suggest to identify material composition and material properties of an arthropod cuticular structure before performing FEM analyses and to increase the validity of the models. Using CLSM for identifying the material composition and targeted nanoindentation in combination with high-resolution X-ray tomography for subsequent quantification will improve the accuracy of FEM and related modelling approaches.

## Data accessibility

Our data can be found in the Dryad Digital Repository (http://dx.doi.org/10.5061/dryad.84n16hg [54]).
